# Systematic Review and Meta-analysis on the Association of Occupational Exposure to Free Crystalline Silica and Rheumatoid Arthritis

**DOI:** 10.1007/s12016-021-08846-5

**Published:** 2021-03-02

**Authors:** A. Morotti, I. Sollaku, F. Franceschini, I. Cavazzana, M. Fredi, E. Sala, G. De Palma

**Affiliations:** 1 Department of Medical and Surgical Specialties, Radiological Sciences and Public Health, Unit of Occupational Health and Industrial Hygiene, University of Brescia, Piazzale Spedali Civili 1, 25121 Brescia, Italy; 2 Rheumatology and Clinical Immunology Unit, ASST Spedali Civili, Brescia, Italy; 3 University Hospital “Spedali Civili Di Brescia,” Rheumatology, Department of Clinical and Experimental Sciences (DSCS), University of Brescia, Brescia, Italy; 4 Unit of Occupational Health, Hygiene, Toxicology and Occupational Prevention, University Hospital “Spedali Civili Di Brescia,”, Brescia, Italy

**Keywords:** Rheumatoid arthritis, Free crystalline silica, Silica, Meta-analysis

## Abstract

• **Occupational exposure to free crystalline silica and tobacco smoking are associated with an increased risk rheumatoid arthritis, with the evidence of an interaction in seropositive subjects**.

• **Further studies in the field are needed to support such association**

We carried out a systematic search for all published epidemiological studies concerning the association between occupational exposure to free crystalline silica (FCS) and subsequent development of rheumatoid arthritis (RA). A meta-analysis was conducted on relevant studies. We searched PubMed and Embase, search engines, for original articles published (from 1960 to November 2019) in any language. In addition, we also searched reference lists of included studies manually for additional relevant articles. Finally, twelve studies were included in the meta-analysis (seven case-control cases and five cohort studies). The odds risks and 95% confidence interval (CI) were calculated using a random effect meta-analysis. A primary meta-analysis (using a random effect model)—regarding RA risk in subjects exposed to FCS—yelled to an overall OR of 1.94 (95% CI 1.46–2.58). We also conducted three further meta-analysis, taking into account the presence of autoantibodies (anti-RF or anti-ACPA) and smoking habits and found a significant association between FCS and RA in both seropositive and seronegative subjects (OR 1.74, 95% CI 1.35–2.25 and OR 1.23, 95% CI 1.06–1.4, respectively) and in seropositive subjects which were smokers (OR 3.30, 95% CI 2.40–4.54). The studies that have investigated the association between RA and occupatational exposure to FCS are still scarce and the heterogeneity between the studies remains high. Some critical limitations have been identified within studies, among which, the methods for assessing exposure stand out. Although with due caution, our results confirm the hypothesis of an association between occupational exposure to FCS and RA development. There was an interaction between FCS and tobacco smoking in RA seropositive workers.

**Supplementary Information:**

The online version contains supplementary material available at 10.1007/s12016-021-08846-5.

## Introduction

The aim of this review and meta-analysis is to investigate possible associations between occupational exposure to free crystalline silica (FCS) and rheumatoid arthritis (RA). Although in the last decades this association has been discreetly studied by epidemiological studies, to the best of our knowledge, this is the first systematic review and meta-analysis to date. The studies conducted to date have led to variable results, motivating our study to carefully seek consistent evidence on a possible association.

RA is a chronic inflammatory disorder that typically affects small and medium-sized joints. The primary lesion is synovitis, with the invasion of immune cells in a normally acellular synovium, leading to the formation of inflammatory “pannus.” This hyperplastic tissue causes cartilage breakdown, bony erosion, and, ultimately, loss of function.

While the main target is represented by the joints, RA can also present itself with a great variety of systemic and extra-articular manifestations: assecondary Sjogren’s syndrome (i.e., xerophthalmia, xerostomia), rheumatoid nodules, haematologic involvement, pericardial and pleural effusions, interstitial lung disease, ocular inflammation, skin vasculitis, etc.[1].

The prevalence in the general population is estimated to be approximately 0.5–1%, and women are two or three times more likely to be affected than men [2].

In the majority of patients with RA, circulating autoantibodies are detectable; the most represented are the rheumatoid factor (RF) and the antibodies to citrullinated protein antigens (ACPA) commonly identified by antibodies to cyclic citrullinated peptides (anti-CCP) [3, 4]. Autoantibodies are part of the ongoing classification criteria, proposed in the 2010 by the collaborative initiative of the American College of Rheumatology (ACR) and European League Against Rheumatism (EULAR) [5].

When one or more of these autoantibodies are detected in serum, RA is defined seropositive (RA+) and the disease might manifest with more severe symptoms such as erosive joint degeneration and rapid radiographic progression [1, 6].

Several studies and multiple scientific evidences have focused in particular on ACPA autoantibodies considered more sensitive and specific for diagnostic and prognostic purposes as early indicators of joint destruction, persistent RA and extra-articular involvement [7, 8].

The main pathogenetic hypothesis about RA development is that of a multifactorial disease, that arises from gene-environment interactions. Among environmental risk factors, the main suspects are for cigarette smoke, FCS, and viral infections [1]. Although the molecular pathogenesis of RA remains to date not fully understood, an increasing number of studies hypothesize that the lung might be a possible starting site of immuno-molecular alterations, alternative to joints [9, 10].

This assumption has been hypothesized in particular in ACPA-positive RA [3, 10] that manifests with early inflammatory lung changes, even many years before joint inflammation, suggesting that autoimmunity starts right in the lung [9].

It has been hypothesized that FCS is able to stimulate the immune response by inducing the expression of citrullinated proteins in lungs. Such modified proteins are presented by MHC complexes expressed on the surface of antigen presenting cells (APCs), that activate T cells, which in turn will stimulate the maturation of B cells responsible for the production of ACPAs [9, 11].

The aim of this study was to evaluate all published literature investigating the association between occupational exposure to FCS and RA development.

## Methods

PRISMA checklist [14] can be found in the supplementary material (Table S3). An a priori* protocol* was defined, in which we established the criteria for inclusion and exclusion of epidemiological studies. We searched PubMed and Embase for original articles published between 1960 and November 2019. The bibliographic search has been extended also using the links “related article” of PubMed and the reference lists of some key studies. There were no restrictions regarding the language of the articles.

We performed the following literature search on PubMed and Embase (Table 1):

**Table 1:** Literature search strategy

Database	Search strategy
PubMed	(cohort studies[mesh:noexp] OR followup studies[mesh:noexp] OR prospective studies[mesh:noexp] OR retrospective studies[mesh:noexp] OR cohort[TIAB] OR prospective[TIAB] OR retrospective[TIAB] OR “Case-Control Studies”[Mesh:noexp] OR "retrospective studies"[mesh:noexp] OR “Control Groups”[Mesh:noexp] OR (case[TIAB] AND control[TIAB]) OR (cases[TIAB] AND controls[TIAB]) OR (cases[TIAB] AND controlled[TIAB]) OR (case[TIAB] AND comparison*[TIAB]) OR (cases[TIAB] AND comparison*[TIAB]) OR “control group”[TIAB] OR “control groups”[TIAB] OR occupational diseases [MH] OR occupational exposure [MH] OR occupational exposure* [TW] OR “occupational health” OR “occupational medicine” OR work-related OR working environment [TW] OR at work [TW] OR work environment [TW] OR occupations [MH] OR work [MH] OR workplace* [TW] OR workload OR occupation* OR worke* OR work place* [TW] OR work site* [TW] OR job* [TW] OR occupational groups [MH] OR employment OR worksite* OR industry) AND (“Rheumatoid Arthritis” OR RA)
Embase	('rheumatoid arthritis'/exp OR 'rheumatoid arthritis' OR 'ra' OR ra) AND ('silica'/exp OR 'silica') OR 'silica'/exp OR silica) AND ('case control study'/exp OR 'case control study') OR 'cohort analysis'/exp OR 'cohort analysis' OR 'observational study'/exp OR 'observational study' OR 'prospective study'/exp OR 'prospective study' OR 'retrospective study'/exp OR 'retrospective study'

PubMed search strategy was developed through the strings proposed by Professor S. Mattioli [12] (occupational diseases [MH] OR […]) and those from the University of Texas School of Public Health Library[13] (“Case–Control Studies "[Mesh: noexp] […]" cohort studies "[mesh: noexp] […]). Subsequently, the following “search filter” was applied to the string: silica.

We performed an additional literature search on Embase.

To be included in the meta-analysis, studies had to fulfill the following criteria: (i) studies examining the association between occupational exposure to FCS and RA; (ii) studies in which a measure of association such as relative risk (RR), odds ratio (OR), standardized mortality ratios (SMR), or standardized incidence ratios (SIR) was either reported or could be derived from data reported in the article. Exclusion criteria were as follows: (i) by study design: experimental studies, case reports, and reviews; (ii) data incompleteness, e.g., presence of prevalence data in only one of the two samples; (iii) use of data from samples of subjects already used in previous studies (in order to avoid duplication of results); (iv) use of non-standardized methods for diagnosis and/or very high risk of inaccuracy in the assessment; and (v) Inadequacy of results for meta-analysis purposes, e.g., studies with insufficient relative risk and CI information.

Two authors (AM, IS) independently reviewed the studies retrieved from the databases, paying particular attention to the inclusion and exclusion criteria. Doubtful cases or disagreement situations were resolved involving a third author (SC).

The final selection of the articles included in the study was based on a careful reading and analysis of the entire texts.

**Fig. 1:**
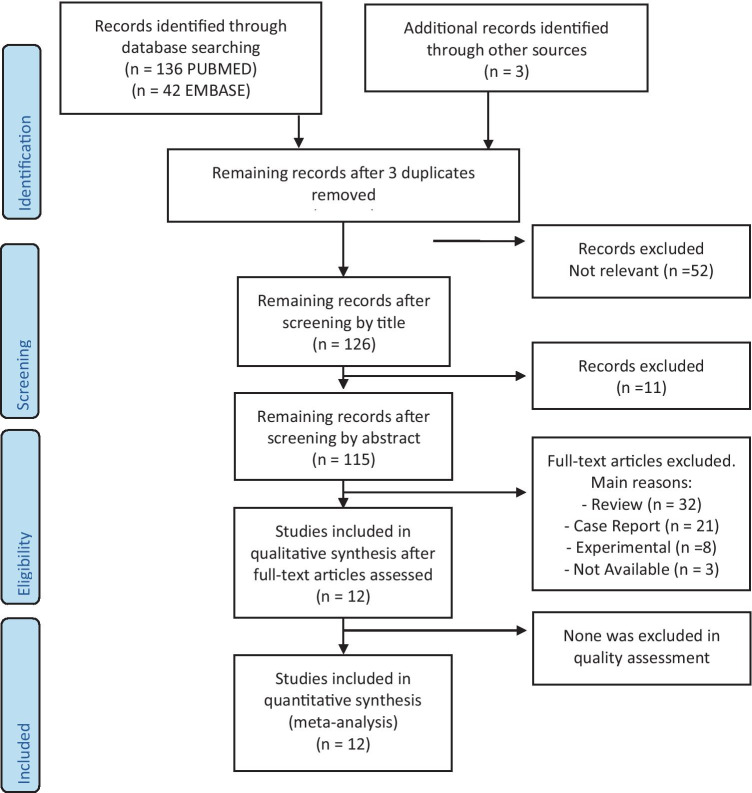
Flow chart of search and selection of studies included in the review and meta-analysis

The whole process followed during the systematic review is shown in Fig. 1. To draw up the flow chart, we used the model on the official PRISMA website [14]. The bibliographic search (through PubMed and Embase) gave rise to 178 articles; three further articles have been identified through other sources [21, 28, 29].

**Fig. 2:**
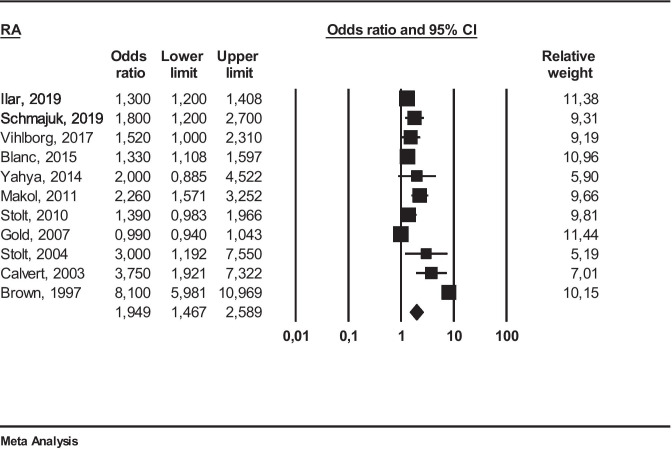
Meta-analysis of all the selected studies on occupational exposure to FCS and RA

Following the title revision, we eliminated 52 articles and further 11 after abstract revision. Additional articles (32 Reviews, 21 Case Reports, 8 Experimental, and 3 not available) were excluded based on the reading of the full text and non-compliance with the inclusion criteria.

Finally, the studies included in qualitative synthesis were twelve, and all of them were eligible for the meta-analysis.

In order to perform the meta-analysis, we extracted the most relevant data from each study (Table 2), including type of exposure, exposure assessment, no. of cases, and effect size (95% CI).

**Table 2:** RA studies included in the Systematic Review and meta-analysis

Study ID	Author	Year	Country	Year	Design	Type of exposure	Exposure assessment	No. of observed cases	Effect size (95% CI)
1	Ilar [18]	2019	Sweden	1996–2013	CC	Occupational – not better specified	JEMs	742 cases/5235 controls (678 men; 64 women)	Any RA: OR 1.3 (1.2–1.5) RA+ : RR 1.28 (1.02–1.61) RA−: RR 1.46 (1.03–2.07)
2	Schmajuk [25]	2019	USA	2019	CO	Mainly coal mining work	Exposure data were self-reported during a telephone questionnaire	556 silica-exposed male workers	Any RA: OR 2.1 (1.1–3.9)
3	Vihlborg [26]	2017	Sweden	1930–2013	CO	Iron foundries	A mixed model was used to calculate silica exposure, and individual silica exposures were used to compute dose responses	2187 silica-exposed male workers	SIR 2.59 (1.24–4.76)
4	Blanc [27]	2015	Sweden	1997–2010	CO	Construction work	JEMs	195 silica-exposed male workers	Any RA: RR 1.33 (1.11–1.60) RA+ : RR 1.28 (1.02–1.61) RA−: RR 1.46 (1.03–2.07) Smoking : Any RA: RR 1.99 (1.66–2.40) RA+ : RR 2.41 (1.89–3.07) RA−: RR 1.52 (1.10–2.12)
5	Yahya [19]	2014	Malaysia	2005–2009	CC	Stone dust, rock drilling, stone crushing	In-person interview according to an extensive questionnaire The questions concerning silica exposure covered time aspects of exposure (when and how long) as well as exposure intensity	14 cases/12 controls	Any RA: OR 2.0 (0.9–4.6) RA+ : OR 2.4 (1.0–5.6) RA−: OR 0.9 (0.2–4.5) *SMOKING:* RA+ : OR 7.5 (2.3 -24.2)
6	Makol [28]	2011	USA	1985–2006	CO	Various including foundry work and sandblasting	30-45 min telephone interview (if the individual was deceased, a next-of-kin was interviewed). Addition medical records, radiographs, laboratory data were also collected	1022 cases diagnosed with Silicosis. (only for the outcome: 24 case of SLE)	Any RA: RR 2.26 (1.57–3.25)
7	Stolt [20]	2009	Sweden	1996–2006	CC	Stone dust, rock drilling, stone crushing	Exposure data were self-reported using a questionnaire	80 cases/69 controls	Any RA: OR 1.39 (0.98–1.96) RA+ : OR 1.67 (1.13–2.48) RA−: OR 0.98 (0.57–1.66) Smoking : Any RA: OR 2.35 (1.46–3.80) RA+ : OR 4.08 (2.31–7.21) RA−: OR 1.16 (0.56–2.39)
8	Gold [21]	2007	USA	1984–1999	CC Mortality-Death certificates data	Among 509 different jobs mainly hand painting, hand coating and hand decorating occupations	JEMs	35,730 cases/260,632 controls	Any RA: OR 0.99 (0.94–1.03)
9	Stolt [22]	2004	Sweden	1996–2001	CC	Stone dust, rock drilling, stone crushing	Exposure data were self-reported using a questionnaire	21 cases/11 controls	Any RA: OR 3.0 (1.2–7.6) RA+ : OR 3.5 (1.1–11.2) RA−: OR 1.7 (0.3–9.3) Smoking : Any RA: OR 3.7 (1.7–8.1) RA+ : OR 5.4 (2.1–14.0) RA−: OR 1.6 (0.4–7.2)
10	Calvert [23]	2003	USA	1982–1995	CC Mortality-Death certificates data	Occupational exposure to FCS not better specified (in general mining and dusty trades)	JEMs	15 cases/20 controls	Any RA: OR 3.75 (1.92–7.32)
11	Brown [29]	1997	Sweden-Denmark	1965–1983	CO Mortality-Death certificates data	A review of Swedish computerized hospital diagnoses with diagnostic codes for both silicosis and SLE. The type of exposure was not better specified	A review of Swedish computerized hospital diagnoses with diagnostic codes for both silicosis and SLE. The exposure assessment was not better specified	57 cases (only for the outcome: 44 cases of SLE)	Any RA: RR 8.1 (5.9–10.82)
12	Sluis-Cremer [24]	1986	South Africa	1967–1979	CC	Gold mines	JEMs	96 cases/157 controls	RA+ : OR 5 (1.99–12.56) RA−: OR 1.44 (0.44–4.73) NOT TESTED: OR 2.25 (0.78–6.43)

*CC* case control, *JEM* job-exposure matrix

The quality of the studies was evaluated by applying the Newcastle Ottawa Scale (NOS)[15], that is available in the Supplementary material (Supplementary Data S1 and S2). Total final quality scores of individual studies are summarized in Supplementary Table S2.

The data were analyzed using the *Comprehensive Meta-analysis v3.0* software from *Biostat Inc.* [16]. We chose to use the random effect model for the aggregate estimation of the association rather than the fixed effect model according to the hypothesis that it was likely to assume a high level of variability among studies. Heterogeneity among studies was assessed using the *I*^2^ index. Heterogeneity was considered *low* if *I*^2^ values were between 25 and 50%, *moderate* if between 50 and 75%, and *high* if higher than 75% [17].

The Funnel Plot was used to identify and estimate the amount of publication bias, whereas the statistical evaluation was carried out by Egger’s test.

## Results

After an accurate procedure of selection and evaluation of 178 studies, the following twelve were deemed as relevant (Table 2):

-7 case-control studies [18–24].

-5 cohort studies [25–29].

### Study Characteristics

Population. In the case-control studies, the cases were classified as RA according to the ACR/EULAR classification criteria (American College of Rheumatology) [19, 20, 22] or the American Rheumatism Society (ARA) criteria[26]. In the case-control mortality studies [21, 23], subjects were identified through the specific death cause code ICD-9 (International Classification of Diseases, 9th revision) in their respective death certificates.

With regard to cohort studies, when declared, subjects with RA were selected using the International Classification of Diseases, 10th revision (ICD-10) [25–27] with *M05* codes for seropositive rheumatoid arthritis and *M06* for seronegative.

#### Exposures

Most of the population studied was dusty trade workers subject to moderate or high occupational exposure to FCS.

#### Controls

Subjects were randomly selected and frequency-matched to the cases by the principal indicators (e.g., age, sex).

#### Outcome

May the occupational exposure to FCS be a significant risk factor in the development of RA? [18–20, 22, 24–29]. May the RA mortality rate possibly be related to occupational exposure to FCS? [21, 23]. The studies were published between 1986 and 2017.

Exposure to FCS concerns different jobs (mainly “dusty trades”). The examined populations are predominantly men. Smoking habit was recorded and studied in four of the studies included in the meta-analysis.

### Risk of Bias Within Studies

The quality and risk of bias appraisal was conducted using the Newcastle Ottawa Scale (NOS)[15] by two independent evaluators (*AM, IS*). We used a modified version of this tool in order to better adapt it to the studies we reviewed (see Supplementary Data S1 and S2).

An overview of the risk of bias for each of the studies included in this work is shown in Supplementary Table 2.

The NOS produced a final score of 7 [18], 6 [19], 8 [20], 7 [22], 5 [24], 7 [26], 4 [27], 3 [21], 4 [23], 5 [25], 7 [28], and 2 [29].

A few study in particular [21, 23, 27, 29] scored low on NOS scale, with significant shortcomings in both case selection and exposure assessment.

We found that the exposure assessment in all the studies has some critical limitations; this can be attributed to the use of self-completed questionnaires or a face-to-face interview in which the patient is asked to remember events dating many years in the past.

A widely used tool is the Job Exposure Matrix (JEM) [30] to estimate exposure to silica-based on work tasks, exposure measurements, and information on the work process[21, 23, 24, 27].

It should also be taken into account that personal Interviews might be subjected to recall biases. The exposure assessment in the case-control mortality studies [21, 23] is particularly weak, as the job and duration was inferred from the death certificate. These are often incomplete and inaccurate. It is also conceivable that the reported employment on the death certificate is attributable to the last job or to that mainly carried out with the risk of misclassification.

Cases of the different studies can be defined as dusty trade workers affected by RA and defined according to the American College of Rheumatology criteria (ACR). In this regard, it is important to note that over years, the classification criteria have undergone different revisions: in 1987 [18–20, 22] and in 2010 (ACR/EULAR criteria) [25].

The 1987 revised ACR classification criteria have been criticized for including late manifestations (e.g., rheumatoid nodules and radiological damage) failing to identify patients with early inflammatory arthritis. Nevertheless, they are able to classify established RA with high sensitivity and specificity.

With regard to cohort studies and mortality studies, the diagnosis of RA was defined according to the International Classification of Diseases (ICD) 10th [26, 27] and 9th Edition [21, 27]. ICD-10 is a classification introduced in 2003 and with greater specificity as compared with ICD-9. The specific RA codes used in ICD-9 were 714.0, 714.1, and 714.2 and in ICD-10 M06.9, M05 for seropositive rheumatoid arthritis and M06 for seronegative rheumatoid arthritis.

A non-negligible limit of the studies that use the ICD classification is to define RA+ patients based on the ICD-M05 code without distinction between antibodies for rheumatoid factor or citrullinated peptide.

In one study [25], the diagnosis of RA was self-reported by cases during the telephone interview, therefore with a high risk of misclassification or reporting bias.

All the studies controlled for the main and potential confounding agents (age, sex, geographical area) with a few exception [21, 29].

Several studies [32–34], reported a strong association between tobacco smoking and RA; however, only a few of the studies adjusted the OR for potential confounding from smoking [19, 20, 25, 27].

Some studies have also investigated the correlation between ever/never smokers exposed to FCS and RA [19, 22, 25, 27].

As usual, in these kinds of studies, participants were selected on a voluntary basis and this may introduce a *selection bias*.

Some main biases affected the identification of cases and controls; specifically, in the Schmajuk et al. study [25], patients were asked to self-report the diagnosis of their physicians and the possible intake of glucocorticoid treatment. In Yahya et al. study [19], both hospital and general population controls have been enrolled.

In the studies [21, 24] enrolling mine workers for long periods, we are aware that such individuals have a greater chance of multiple diseases and therefore false associations are more likely to report.

### Synthesis of Results

#### Primary Meta-Analysis Results

he meta-analysis of eleven studies [18–23, 25–29], applying a random effect model, yielded an overall OR of 1.94 (95% CI 1.46–2.58) with *I*^2^ = 95% (pronounced heterogeneity). This result was statistically significant (*p* ≤ 0.05).

#### Subgroup Analysis Results (By Autoantibodies)

We performed an additional meta-analysis on seven studies[18–20, 24–27], using a random effect, to investigate the relationship between occupational exposure to FCS and the effect on seropositive (RA+) or seronegative (RA−) RA patients, either for ACPA and/or RF (Fig. 2).

**Fig. 3:**
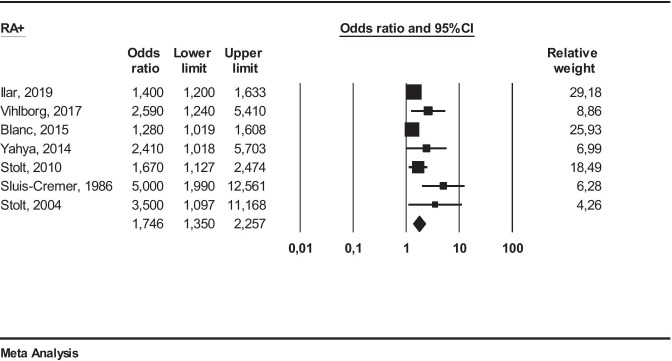
Meta-analysis of studies including seropositive (RA+) RA patients

*RA*+ *patients*: We obtained the following result: OR 1.74 (95% CI 1.35–2.25 (*I*^2^ = 59%)). This result was statistically significant (*p* ≤ 0.05) (Fig. 3).

**Fig. 4:**
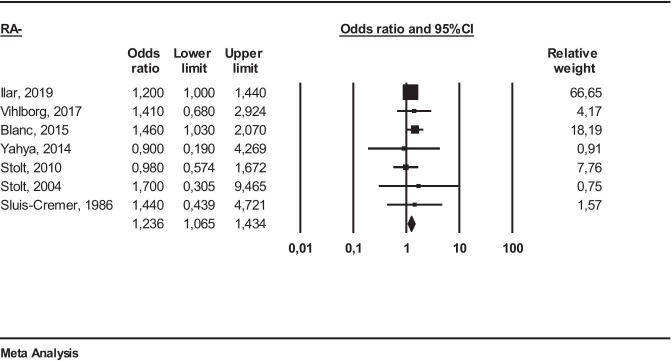
Meta-analysis of studies including seronegative (RA−) RA patients

*RA−*: We obtained the following result: (OR 1.23 (95% CI 1.06–1.43) (*I*^2^ = 0%)). Significant association has been observed (*p* ≤ 0.05) (Fig. 4).

#### Subgroup Analysis Results (By Smoking)

**Fig. 5:**
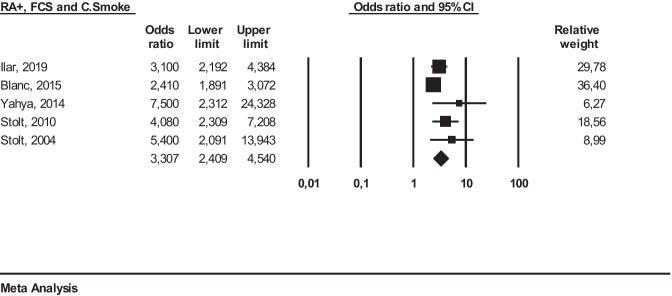
Meta-analysis of studies investigating interaction between occupational exposure to FCS, smoking habits, and seropositive (RA+) RA patients

Finally, we performed a further meta-analysis on five studies [18–20, 25, 27] to investigate the relationship between smoking habits and occupational exposure to FCS and the effect on RA+ development (Fig. 5).

Using a random effect, we obtained the following result: OR 3.30 (95% CI 2.40–4.54 (*I*^2^ = 49%)). This result was again statistically significant (*p* ≤ 0.05).

### Risk of Bias Across the Studies (Funnel Plot)

We used the Funnel Plot, Egger’s test, and Higgins index to detect possible biases between the studies included in the meta-analysis (Supplementary Figures S6-S9).

Using the Higgins *I*^2^ index, we obtained the following results:

*Any RA* meta-analysis: *I*^2^ = 95% (Figure S6), *RA*+ meta-analysis: *I*^2^ = 59% (Figure S7), *RA−* meta-analysis: *I*^2^ = 0% (Figure S8) and *RA*+ *, FCS and cigarette smoke* meta-analysis: *I*^2^ =  49,% (Figure S9).

The visible asymmetry in the Funnel Plots confirms the presence of heterogeneity in the majority of the meta-analyses. The *p*-value of the Egger’s regression test resulted significant (*p* < 0.05) for publication bias, in all the performed meta-analyses.

## Discussion

### Summary of Evidence

To the best of our knowledge, the present study represents the first meta-analysis that analyzes the correlation between development of RA and occupational exposure to FCS.

Since the status of seropositive/seronegative RA subjects has assumed increasingly prognostic importance, we performed two additional meta-analysis, one for each subgroup.

Furthermore, some epidemiological studies [35–37] have shown that lifelong cigarette smoking is associated with an increased risk of RA, particularly for ACPA positive subjects [38]. According to the main pathogenetic hypothesis, smokers inhale a lot of irritant compounds[39], that may act as triggers of the immune system. FCS could act like smoking[20]; therefore, we performed a meta-analysis to investigate a possible synergy between both risk factors.

#### Main Meta-Analysis

Six out of twelve studies included in this systematic review were performed in Sweden. Three [18, 20, 31] were based on the same extensive population-based study EIRA (Epidemiological Investigation of Rheumatoid Arthritis) [40]. The others made use as well of national registers. We can speculate that the predominance of Swedish studies might be due to national regulations that require constant checkups for workers occupationally exposed to FCS and a valid National Register of RA patients.

The remaining studies were performed in the USA [21, 23, 25, 28], South Africa [24], and Malaysia [19].

All studies have long-running data sets conducted on workers mainly involved in *dusty trades*, including also iron foundries [26] and coal or gold mines [25, 26].

The results of our study support the pathogenetic hypothesis that occupational exposure to FCS is a possible risk factor for RA. The final magnitude of the association obtained from the primary meta-analysis is statistically significant but we have found a high heterogeneity among studies ((OR 1.94 95% CI 1.46–2.58) *I*^2^ = 95%). Such heterogeneity can be explained by different study designs, studied populations, and exposure assessment.

It is important to highlight that four studies [23, 24, 28, 29] include silicotic patients. We believe that such studies are particularly valuable because they give us the certainty about occupational exposure to FCS. Silicosis is indeed a progressive and irreversible disease caused by inhaling large amounts of FCS, usually over decades (10–20 years) [41].

We specify that in the study Brown et al. [29] for the purpose of meta-analysis, we decided to extract the most conservative risk index. Furthermore, it is specified that the Sluis-Cremer et al., 1986 study [24] was also excluded from the meta-analysis (FCS-RA) due to the lack of an appropriate OR. The study was instead included in the subgroup meta-analysis since it provided ORs for RF+ and RF− RA patients. We could not perform analyzes stratified by age since only one study[22] provided age groups.

#### Meta-Analysis of the Autoantibodies

With this further analysis, we investigated the association between exposure to FCS and seropositive/seronegative RA.

Several studies have shown that autoantibodies may be originated in the lungs by autoimmune responses to citrullinated peptides. Moreover, it is well known that anti-CCP is more sensitive than RF and may appear earlier in the course of RA[42]. Only few studies have provided data about autoantibodies, and among these, few have specified the type of autoantibody: RF and ACPA [19, 27], ACPA [20], and RF [24]. Cases were considered RA+ , if including subjects characterized by positivity for rheumatoid factor and/or anti-CCP (ACPA) antibodies. The corresponding OR observed in these meta-analyzes for subgroups were as follows: for RA+ : 1.74 (95% CI 1.35–2.25) while for RA−: 1.23 (95% CI 1.06–1.4).

These results indicate that the association between occupational exposure to FCS and RA is higher for seropositive RA subjects. Nevertheless, such results are yet based on few studies and with limited numbers of cases to consider them as conclusive.

#### The Role of Tobacco Smoking

Tobacco smoking is considered a main risk factor for ACPA+ RA development. The pathogenic mechanism is still unknown, and it is not clear how the breakdown of immunological tolerance towards citrullinated proteins and the production of ACPAs occurs [43].

Since both FCS and tobacco smoke are inhaled through the airways and are deposited at the lung level, it has been speculated that both can act synergistically in the establishment of RA[10]. To perform this meta-analysis, we considered *ever smokers*/*RA*+ cases who had been occupationally exposed to FCS. The OR we obtained was 3.30 (95% CI 2.40–4.50). Such result reinforces the hypothesis of a synergistic interaction between cigarette smoking and occupational exposure to FCS. Nevertheless, the limited number of studies included in this meta-analysis as well as the presence of further unknown confounding agents implies caution in the interpretation of the results

### Limitations

A major limitation of meta-analysis, and this one is not exempt, is that the investigated populations, the FCS exposure, and the outcome definitions are not the same across the studies.

As evidenced by the use of the Newcastle and Ottawa scale, the quality of the studies is comparable and attested with acceptable values. However, the adjustment of the main confounding factors and the response rate of cases and controls, obtained different grades in each study (Supplementary Table S2).

This study is not exempt by Publication bias as shown in asymmetry of the Funnel Plot.

## Conclusions

To the best of our knowledge, the present study represents the first systematic review and meta-analysis performed, investigating such association.

Our study, notwithstanding some limitations, expands knowledge about the FCS-RA association with greater strength than the single studies published so far.

The results of our meta-analysis confirm an increased risk of rheumatoid arthritis, especially if seropositive, among subjects with previous significant occupational exposure to FCS. With an additional meta-analysis, we proved a solid interaction between smoking habits and occupational exposure FCS, mostly in RA seropositive patients.

However, it must be remembered that to date, evidence has not conclusively demonstrated that silica is a causative factor of RA.

As discussed previously, some critical limitations have been identified within studies, among which, the methods for assessing exposure stand out.

More efforts are needed to collect more accurate exposure assessments and limit the heterogeneity among studies. If desirable, that further case-report and cohort studies with a sufficient number of subjects will be conducted in the near future.

## Supplementary Information

Below is the link to the electronic supplementary material.

Supplementary file1 (DOCX 73 KB)

## Abbreviations

FCS: Free crystalline silica
RA: Rheumatoid arthritis
NOS: Newcastle Ottawa Scale
CC: Case-control
CI: Confidence interval
PRISMA: Preferred Reporting Items for Systematic Reviews and Meta-Analyses
OR: Odds ratio
