# Supplementary material for: Systematic Review and Meta-analysis on the Association of Occupational Exposure to Free Crystalline Silica and Rheumatoid Arthritis
Source: Clin Rev Allergy Immunol. 2021 Mar 2;62(2):333–45. doi: 10.1007/s12016-021-08846-5 (PMC8994741; doi:10.1007/s12016-021-08846-5)
Supplement: Supplementary file 1 — Supplementary file1 (DOCX 73 KB) [file 12016_2021_8846_MOESM1_ESM.docx]

SUPPLEMENTARY MATERIALS

**Supplementary Figure S6. *Funnel Plot (any RA)***

**Supplementary Figure S7. *Funnel Plot (RA+)***

**Supplementary Figure S8. *Funnel Plot (RA-)***

**Supplementary Figure S9. *Funnel Plot (RA+, FCS and Cigarette smoke)***

**SUPPLEMENTARY DATA S1
NEWCASTLE - OTTAWA QUALITY ASSESSMENT SCALE**

**CASE CONTROL STUDIES**

Note: A study can be awarded a maximum of one star for each numbered item within the Selection and Exposure categories. A maximum of two stars can be given for Comparability.

**Selection**

1) Is the case definition adequate?

a) yes, with independent validation **🟑**

b) yes, e.g. record linkage or based on self reports

c) no description

2) Representativeness of the cases

a) consecutive or obviously representative series of cases **🟑**

b) potential for selection biases or not stated

3) Selection of Controls

a) community controls **🟑**

b) hospital controls

c) no description

4) Definition of Controls

a) no history of disease (endpoint) **🟑**

b) no description of source

**Comparability**

1) Comparability of cases and controls on the basis of the design or analysis

a) study controls for _______________ (Select the most important factor.) **🟑**

b) study controls for any additional factor **🟑** (This criteria could be modified to indicate specific control for a second important factor.)

**Exposure**

1) Ascertainment of exposure

a) secure record (e.g. surgical records) **🟑**

b) structured interview where blind to case/control status **🟑**

c) interview not blinded to case/control status

d) written self report or medical record only

e) no description

2) Same method of ascertainment for cases and controls

a) yes **🟑**

b) no

3) Non-Response rate

a) same rate for both groups **🟑**

b) non respondents described

c) rate different and no designation

**SUPPLEMENTARY DATA S2
NEWCASTLE - OTTAWA QUALITY ASSESSMENT SCALE - COHORT STUDIES**

Note: A study can be awarded a maximum of one star for each numbered item within the Selection and Outcome categories. A maximum of two stars can be given for Comparability

**Selection**

1) Representativeness of the exposed cohort

a) truly representative of the average _______________ (describe) in the community **🟑**

b) somewhat representative of the average ______________ in the community **🟑**

c) selected group of users eg nurses, volunteers

d) no description of the derivation of the cohort

2) Selection of the non-exposed cohort

a) drawn from the same community as the exposed cohort **🟑**

b) drawn from a different source

c) no description of the derivation of the non-exposed cohort

3) Ascertainment of exposure

a) secure record (eg surgical records) **🟑**

b) structured interview **🟑**

c) written self-report

d) no description

4) Demonstration that outcome of interest was not present at start of study

a) yes **🟑**

b) no

**Comparability**

1) Comparability of cohorts on the basis of the design or analysis

a) study controls for _____________ (select the most important factor) **🟑**

b) study controls for any additional factor **🟑** (This criteria could be modified to indicate specific control for a second important factor.)

**Outcome**

1) Assessment of outcome

a) independent blind assessment **🟑**

b) record linkage **🟑**

c) self-report

d) no description

2) Was follow-up long enough for outcomes to occur

a) yes (select an adequate follow up period for outcome of interest) **🟑**

b) no

3) Adequacy of follow up of cohorts

a) complete follow up - all subjects accounted for **🟑**

b) subjects lost to follow up unlikely to introduce bias - small number lost - > ____ % (select an adequate %) follow up, or description provided of those lost) **🟑**

c) follow up rate < ____% (select an adequate %) and no description of those lost

d) no statement

**Supplementary Table S2. Results of quality assessment of the papers included in the meta-analysis of FCS exposure and risk of RA (NOS)**

| **Case-Control studies** | | | | | | | | | |
| --- | --- | --- | --- | --- | --- | --- | --- | --- | --- |
|  | **Selection** | | | | **Comparability** | **Exposure** | | | |
| **Study** | **Is the case definition adequate?** | **Representativeness of the cases** | **Selection of Controls** | **Definition of Controls** | **Comparability of cases and controls on the basis of the design or analysis** | **Ascertainment of exposure** | **Same method of ascertainment for cases and controls** | **Non-response rate** | **Score** |
| **Ilar et al., 2019** | * | * | * | * | ** | **/** | * | **/** | **7** |
| **Yahya et al., 2014** | * | * | **/** | * | ** | **/** | * | **/** | **6** |
| **Stolt et al., 2009** | * | * | * | * | ** | **/** | * | * | **8** |
| **Gold et al., 2007** | **/** | **/** | * | * | **/** | **/** | * | **/** | **3** |
| **Stolt et al., 2005** | * | * | * | * | ** | **/** | * | **/** | **7** |
| **Calvert et al., 2003** | **/** | **/** | **/** | * | ** | **/** | * | **/** | **4** |
| **Sluis-Cremer et al., 1986** | * | * | **/** | * | * | **/** | * | **/** | **5** |

| **Cohort studies** | | | | | | | | | |
| --- | --- | --- | --- | --- | --- | --- | --- | --- | --- |
|  | **Selection** | | | | **Comparability** | **Outcome** | | | |
| **Study** | **Representativeness of the exposed cohort** | **Selection of the non-exposed cohort** | **Ascertainment of exposure** | **Demonstration that outcome of interest was not present at start of study** | **Comparability of cohorts on the basis of the design or analysis** | **Assessment of outcome** | **Was follow-up long enough for outcomes to occur** | **Adequacy of follow up of cohorts** | **Score** |
| **Schmajuk et al. 2019** | * | **/** | * | * | * | **/** | * | **/** | **5** |
| **Vihlborg et al., 2017** | * | * | * | **/** | * | * | * | * | **7** |
| **Blanc et al., 2015** | * | **/** | * | **/** | **/** | **/** | * | * | **4** |
| **Makol et al. 2011** | * | * | * | * | **/** | * | * | * | **7** |
| **Brown et al. 1997** | **/** | **/** | * | **/** | **/** | * | **/** | **/** | **2** |

*Note: *,**: positively evaluated items (* =1; **=2), according to the Newcastle-Ottawa scale*

*/ : not evaluable items*

**Supplementary Table S3. Prisma Checklist**

| **Section/topic** | **#** | **Checklist item** | **Reported on page #** |
| --- | --- | --- | --- |
| **TITLE** | | |  |
| Title | 1 | Identify the report as a systematic review, meta-analysis, or both. | 1 |
| **ABSTRACT** | | |  |
| Structured summary | 2 | Provide a structured summary including, as applicable: background; objectives; data sources; study eligibility criteria, participants, and interventions; study appraisal and synthesis methods; results; limitations; conclusions and implications of key findings; systematic review registration number. | 2 |
| **INTRODUCTION** | | |  |
| Rationale | 3 | Describe the rationale for the review in the context of what is already known. | 3 |
| Objectives | 4 | Provide an explicit statement of questions being addressed with reference to participants, interventions, comparisons, outcomes, and study design (PICOS). | 6 |
| **METHODS** | | |  |
| Protocol and registration | 5 | Indicate if a review protocol exists, if and where it can be accessed (e.g., Web address), and, if available, provide registration information including registration number. | 4 |
| Eligibility criteria | 6 | Specify study characteristics (e.g., PICOS, length of follow-up) and report characteristics (e.g., years considered, language, publication status) used as criteria for eligibility, giving rationale. | 4 |
| Information sources | 7 | Describe all information sources (e.g., databases with dates of coverage, contact with study authors to identify additional studies) in the search and date last searched. | 4 |
| Search | 8 | Present full electronic search strategy for at least one database, including any limits used, such that it could be repeated. | 4 |
| Study selection | 9 | State the process for selecting studies (i.e., screening, eligibility, included in systematic review, and, if applicable, included in the meta-analysis). | 4 |
| Data collection process | 10 | Describe method of data extraction from reports (e.g., piloted forms, independently, in duplicate) and any processes for obtaining and confirming data from investigators. | 4 |
| Data items | 11 | List and define all variables for which data were sought (e.g., PICOS, funding sources) and any assumptions and simplifications made. | 4 |
| Risk of bias in individual studies | 12 | Describe methods used for assessing risk of bias of individual studies (including specification of whether this was done at the study or outcome level), and how this information is to be used in any data synthesis. | 4 |
| Summary measures | 13 | State the principal summary measures (e.g., risk ratio, difference in means). | 5 |
| Synthesis of results | 14 | Describe the methods of handling data and combining results of studies, if done, including measures of consistency (e.g., I^2^) for each meta-analysis. | 5 |

Page 1 of 2

| **Section/topic** | **#** | **Checklist item** | **Reported on page #** |
| --- | --- | --- | --- |
| Risk of bias across studies | 15 | Specify any assessment of risk of bias that may affect the cumulative evidence (e.g., publication bias, selective reporting within studies). | 6 |
| Additional analyses | 16 | Describe methods of additional analyses (e.g., sensitivity or subgroup analyses, meta-regression), if done, indicating which were pre-specified. | - |
| **RESULTS** | | |  |
| Study selection | 17 | Give numbers of studies screened, assessed for eligibility, and included in the review, with reasons for exclusions at each stage, ideally with a flow diagram. | 12 |
| Study characteristics | 18 | For each study, present characteristics for which data were extracted (e.g., study size, PICOS, follow-up period) and provide the citations. | 13 |
| Risk of bias within studies | 19 | Present data on risk of bias of each study and, if available, any outcome level assessment (see item 12). | 6 |
| Results of individual studies | 20 | For all outcomes considered (benefits or harms), present, for each study: (a) simple summary data for each intervention group (b) effect estimates and confidence intervals, ideally with a forest plot. | 8 |
| Synthesis of results | 21 | Present results of each meta-analysis done, including confidence intervals and measures of consistency. | 8 |
| Risk of bias across studies | 22 | Present results of any assessment of risk of bias across studies (see Item 15). | 8 |
| Additional analysis | 23 | Give results of additional analyses, if done (e.g., sensitivity or subgroup analyses, meta-regression [see Item 16]). | 8 |
| **DISCUSSION** | | |  |
| Summary of evidence | 24 | Summarize the main findings including the strength of evidence for each main outcome; consider their relevance to key groups (e.g., healthcare providers, users, and policy makers). | 9 |
| Limitations | 25 | Discuss limitations at study and outcome level (e.g., risk of bias), and at review-level (e.g., incomplete retrieval of identified research, reporting bias). | 10 |
| Conclusions | 26 | Provide a general interpretation of the results in the context of other evidence, and implications for future research. | 10 |
| **FUNDING** | | |  |
| Funding | 27 | Describe sources of funding for the systematic review and other support (e.g., supply of data); role of funders for the systematic review. | 11 |

| **Only for Reviewers** | | | | | | | |
| --- | --- | --- | --- | --- | --- | --- | --- |
| **RA** | **OR** | **Lower Limit** | **Upper Limit** | **Confidence level** | **OR** | **Log odds ratio** | **Std Err** |
| Ilar, 2019 | 1,3 | 1,2 |  | 0,95 | 1,3 | 0,262364 | 4,08E-02 |
| Schmajuk, 2019 | 1,8 | 1,2 |  | 0,95 | 1,8 | 0,587787 | 0,206874 |
| Vihlborg, 2017 | 1,52 | 1 |  | 0,95 | 1,52 | 0,41871 | 0,213632 |
| Blanc, 2015 | 1,33 | 1,11 | 1,6 | 0,95 | 1,33 | 0,285179 | 9,33E-02 |
| Yahya, 2014 | 2 | 0,9 | 4,6 | 0,95 | 2 | 0,693147 | 0,416185 |
| Makol, 2011 | 2,26 | 1,57 | 3,25 | 0,95 | 2,26 | 0,815365 | 0,18561 |
| Stolt, 2010 | 1,39 | 0,98 | 1,96 | 0,95 | 1,39 | 0,329304 | 0,176827 |
| Gold, 2007 | 0,99 | 0,94 |  | 0,95 | 0,99 | -1,01E-02 | 2,64E-02 |
| Stolt, 2004 | 3 | 1,2 | 7,6 | 0,95 | 3 | 1,098612 | 0,470883 |
| Calvert, 2003 | 3,75 | 1,92 | 7,32 | 0,95 | 3,75 | 1,321756 | 0,341406 |
| Brown, 1997 | 8,1 | 5,9 | 10,82 | 0,95 | 8,1 | 2,091864 | 0,154708 |
|  |  |  |  |  |  |  |  |
| **RA-** |  |  |  |  |  |  |  |
| Ilar, 2019 | 1,2 | 1 |  | 0,95 | 1,2 | 0,182322 | 9,30E-02 |
| Vihlborg, 2017 | 1,41 | 0,68 |  | 0,95 | 1,41 | 0,34359 | 0,372074 |
| Blanc, 2015 | 1,46 | 1,03 | 2,07 | 0,95 | 1,46 | 0,378436 | 0,178062 |
| Yahya, 2014 | 0,9 | 0,2 | 4,5 | 0,95 | 0,9 | -0,10536 | 0,794279 |
| Stolt, 2010 | 0,98 | 0,57 | 1,66 | 0,95 | 0,98 | -2,02E-02 | 0,272693 |
| Stolt, 2004 | 1,7 | 0,3 | 9,3 | 0,95 | 1,7 | 0,530628 | 0,876033 |
| Sluis-Cremer, 1986 | 1,44 | 0,44 | 4,73 | 0,95 | 1,44 | 0,364643 | 0,605854 |
|  |  |  |  |  |  |  |  |
| **RA+** |  |  |  |  |  |  |  |
| Ilar, 2019 | 1,4 | 1,2 |  | 0,95 | 1,4 | 0,336472 | 0,07865 |
| Vihlborg, 2017 | 2,59 | 1,24 |  | 0,95 | 2,59 | 0,951658 | 0,375796 |
| Blanc, 2015 | 1,28 | 1,02 | 1,61 | 0,95 | 1,28 | 0,24686 | 0,116439 |
| Yahya, 2014 | 2,41 | 1 | 5,6 | 0,95 | 2,41 | 0,879627 | 0,439489 |
| Stolt, 2010 | 1,67 | 1,13 | 2,48 | 0,95 | 1,67 | 0,512824 | 0,200524 |
| Sluis-Cremer, 1986 | 5 | 1,99 | 12,56 | 0,95 | 5 | 1,609438 | 0,470004 |
| Stolt, 2004 | 3,5 | 1,1 | 11,2 | 0,95 | 3,5 | 1,252763 | 0,592002 |
|  |  |  |  |  |  |  |  |
| **RA+, FCS and C.Smoke** | |  |  |  |  |  |  |
| Ilar, 2019 | 3,1 | 2,2 | 4,4 | 0,95 | 3,1 | 1,131402 | 0,176827 |
| Blanc, 2015 | 2,41 | 1,89 | 3,07 | 0,95 | 2,41 | 0,879627 | 0,123752 |
| Yahya, 2014 | 7,5 | 2,3 | 24,2 | 0,95 | 7,5 | 2,014903 | 0,600379 |
| Stolt, 2010 | 4,08 | 2,31 | 7,21 | 0,95 | 4,08 | 1,406097 | 0,290368 |
| Stolt, 2004 | 5,4 | 2,1 | 14 | 0,95 | 5,4 | 1,686399 | 0,483968 |
